# Machine Learning in Aging: An Example of Developing Prediction Models for Serious Fall Injury in Older Adults

**DOI:** 10.1093/geroni/igaa057.859

**Published:** 2020-12-16

**Authors:** Jaime Speiser, Kathryn Callahan, Jason Fanning, Thomas Gill, Anne Newman, Marco Pahor, Jack Rejeski, Denise Houston

**Affiliations:** 1 Wake Forest School of Medicine, Winston-Salem, North Carolina, United States; 2 Wake Forest University, Winston-Salem, North Carolina, United States; 3 Yale University, New Haven, Connecticut, United States; 4 University of Pittsburgh, Pittsburgh, Pennsylvania, United States; 5 University of Florida, Gainesville, Florida, United States

## Abstract

Advances in computational algorithms and the availability of large datasets with clinically relevant characteristics provide an opportunity to develop machine learning prediction models to aid in diagnosis, prognosis, and treatment of older adults. Some studies have employed machine learning methods for prediction modeling, but skepticism of these methods remains due to lack of reproducibility and difficulty understanding the complex algorithms behind models. We aim to provide an overview of two common machine learning methods: decision tree and random forest. We focus on these methods because they provide a high degree of interpretability. We discuss the underlying algorithms of decision tree and random forest methods and present a tutorial for developing prediction models for serious fall injury using data from the Lifestyle Interventions and Independence for Elders (LIFE) study. Decision tree is a machine learning method that produces a model resembling a flow chart. Random forest consists of a collection of many decision trees whose results are aggregated. In the tutorial example, we discuss evaluation metrics and interpretation for these models. Illustrated in data from the LIFE study, prediction models for serious fall injury were moderate at best (area under the receiver operating curve of 0.54 for decision tree and 0.66 for random forest). Machine learning methods may offer improved performance compared to traditional models for modeling outcomes in aging, but their use should be justified and output should be carefully described. Models should be assessed by clinical experts to ensure compatibility with clinical practice.

